# Asian Elephants in China: Estimating Population Size and Evaluating Habitat Suitability

**DOI:** 10.1371/journal.pone.0124834

**Published:** 2015-05-19

**Authors:** Li Zhang, Lu Dong, Liu Lin, Limin Feng, Fan Yan, Lanxin Wang, Xianming Guo, Aidong Luo

**Affiliations:** 1 Key Laboratory for Biodiversity Science and Ecological Engineering, Ministry of Education, College of Life Sciences, Beijing Normal University, Beijing, 100875, China; 2 College of Life Sciences, Hainan Normal University, Haikou, 571158, China; 3 Research Institute of Xishuangbanna National Nature Reserve, Jinghong, 666100, China; Institute of Zoology, CHINA

## Abstract

We monitored the last remaining Asian elephant populations in China over the past decade. Using DNA tools and repeat genotyping, we estimated the population sizes from 654 dung samples collected from various areas. Combined with morphological individual identifications from over 6,300 elephant photographs taken in the wild, we estimated that the total Asian elephant population size in China is between 221 and 245. Population genetic structure and diversity were examined using a 556-bp fragment of mitochondrial DNA, and 24 unique haplotypes were detected from DNA analysis of 178 individuals. A phylogenetic analysis revealed two highly divergent clades of Asian elephants, α and β, present in Chinese populations. Four populations (Mengla, Shangyong, Mengyang, and Pu’Er) carried mtDNA from the α clade, and only one population (Nangunhe) carried mtDNA belonging to the β clade. Moreover, high genetic divergence was observed between the Nangunhe population and the other four populations; however, genetic diversity among the five populations was low, possibly due to limited gene flow because of habitat fragmentation. The expansion of rubber plantations, crop cultivation, and villages along rivers and roads had caused extensive degradation of natural forest in these areas. This had resulted in the loss and fragmentation of elephant habitats and had formed artificial barriers that inhibited elephant migration. Using Geographic Information System, Global Positioning System, and Remote Sensing technology, we found that the area occupied by rubber plantations, tea farms, and urban settlements had dramatically increased over the past 40 years, resulting in the loss and fragmentation of elephant habitats and forming artificial barriers that inhibit elephant migration. The restoration of ecological corridors to facilitate gene exchange among isolated elephant populations and the establishment of cross-boundary protected areas between China and Laos to secure their natural habitats are critical for the survival of Asian elephants in this region.

## Introduction

The Asian elephant (*Elephas maximus*) is the largest terrestrial animal in Asia. It occurs in Bangladesh, Bhutan, Cambodia, China, India, Indonesia, Laos, Malaysia, Myanmar, Nepal, Sir Lanka, Thailand, and Vietnam. The total population size of Asian elephants was approximately 34,470–53,720 across their range [1].

In China, Asian elephants were found only in the remaining fragmented seasonal rain forests and evergreen forests in southern Yunnan Province, bordering Myanmar and Laos. An earlier report estimated that the Chinese elephant population size was approximately 150 in 1976 [2]. Currently, the estimated population size of wild Asian elephants in China was approximately 200 [3]. Habitat alteration and loss, as well as illegal killing, were the main threats to their survival. Zhang and Wang [4] reported that many of the remaining local forests were destroyed for commercial profit before a logging ban was established in 1998, and most of the elephants’ suitable natural habitats were converted to rubber, tea, or other crops beginning in the 1970s. Between 1988 and 2003, eight elephants were found dead in the Nangunhe Nature Reserve, at least three of which were killed for ivory or due to human/elephant conflicts [5]. In addition, from 2002 to 2007, over 30 elephants were killed illegally in the Shangyong Nature Reserve in Xishuangbanna [6].

Habitat selection by Asian elephants had been studied in China and many other regions in Asia over the past three decades [5–12]. Research on the wild elephant in China had been carried out since the 1970s. During that time, the distribution of elephants had been greatly reduced, and elephants were now confined to the Mengyang, Mengla and Shangyong Protected Areas in Xishuangbanna Prefecture; the Nangunhe Protected Area in Lincang Prefecture; and Simao, Lancang and Jiangcheng in Pu’Er City and surrounding areas. This distribution contrasted markedly the large continuous range occupied by elephants in the 1970s and 1980s[13]. Geographical variation in vegetation cover could influence the diet and habitat selection of elephants in different regions [14], and human activities had strongly impacted elephant behaviors, including movements and migration patterns [9,13,15,16].

Previous interview-based surveys had estimated the total number of wild Asian elephants in China at approximately 165 to 213 individuals [11]. However, these estimates were highly unreliable because the dense rainforest habitat made direct observations of individuals and subsequent estimation of population parameters difficult. If the population structure and genetic diversity of elephants throughout China are described, improved conservation strategies can be implemented to protect this endangered species.

The Asian elephant is one of the first category protected species in China, and local minorities used to believe that elephants were symbols of luck. However, farmers did not welcome the arrival of wild elephants to their croplands, areas which decades ago contained elephant habitat [4]. Competition between humans and elephants over the limited natural resources had negatively affected both species. Crop raiding by elephants was frequently reported in communities within and surrounding protected areas in all counties with the species’ range. Elephants targeted sugar cane, corn, pineapples, and oranges, which were major income for locals, and crop loss due to elephants has increased sharply since the early 2000s [11]. Meanwhile, planting rubber and tea as alternative crops had also led to the extensive degradation of the natural habitat.

In the present study, we investigated the Asian elephant population and their habitat status in China via a decade-long monitoring program, enabling a comprehensive conservation strategy to be developed in order to secure the survival of this endangered species in China.

## Materials and Methods

### Research areas

**Fig 1:**
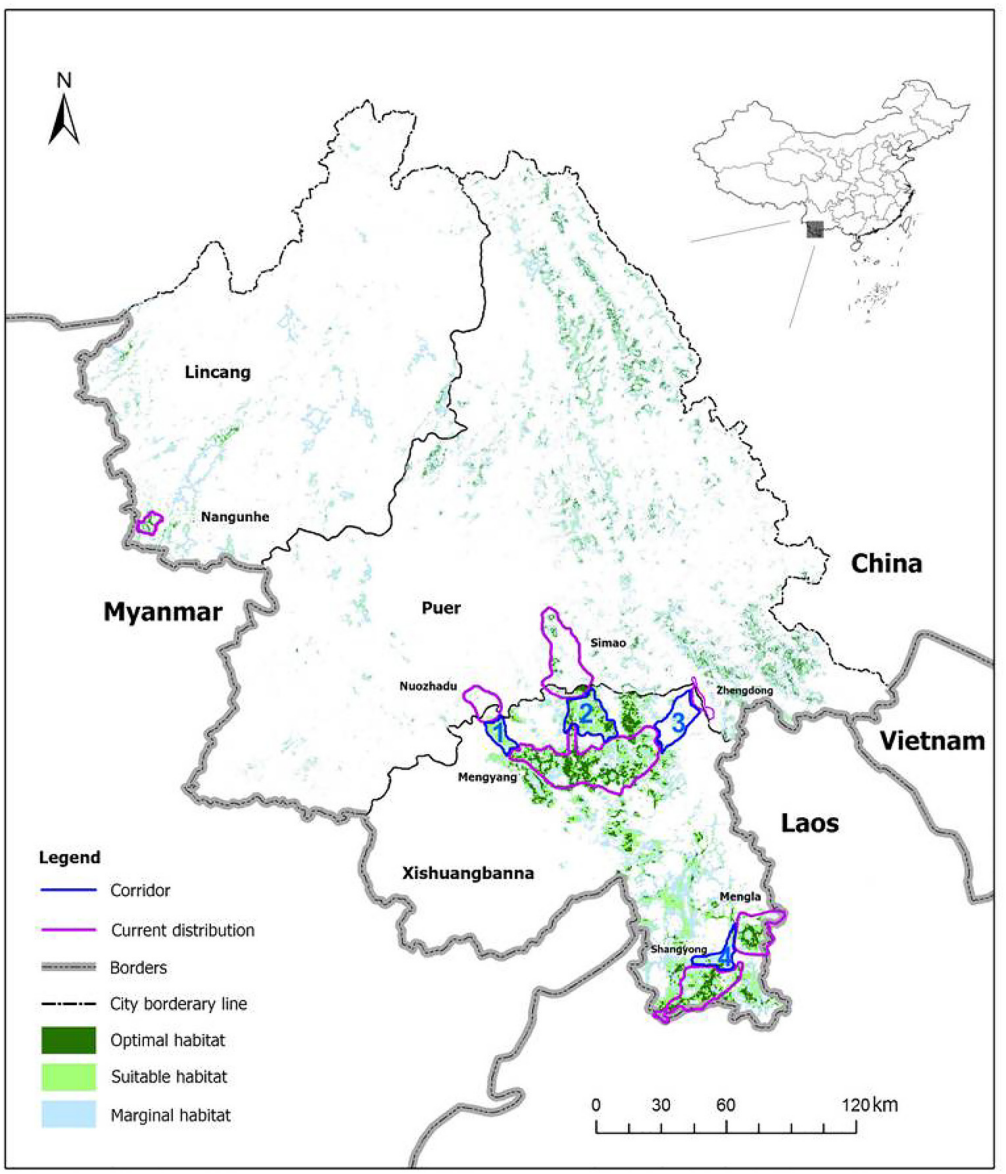
Current distribution of Asian elephants in China with suggested corridors to link isolated populations.

The remaining Asian elephant populations lived in the lush but fragmented rainforests covering the southern Yunnan of China bordering Myanmar and Laos [11]. Our research area covered the following regions between N 21.14°–23.49° and E98.88°–102.36°, including Xishuangbanna (Mengyang-MY, Mengla-ML, and Shangyong-SY), Pu’Er (Simao-SM, Lancang-LC, and Jiangcheng-JC) and Lincang (Nangunhe-NGH) prefectures in southern Yunnan, totaling 38,647.92 km^2^ (Fig 1).

### Identification of individuals

A photographic database was established cataloguing elephant family groups and adult bulls. Individuals were identified by distinguishing ear characteristics, such as cuts, holes, and degree of folding; tusks characteristics (in males), including size, shape, and presence of broken tips; and other morphological characteristics [17]. From 1999 to 2011, over 6,300 elephant photos were added to the database. We also recorded reports of elephant sightings from local villagers. Upon receiving such a report, a member of our research group would visit the site as soon as possible and interviewed local villagers and forest rangers to collect data on the number of individual elephants, the age composition of the group, and their direction of travel. The locations of all identified individuals and family groups were recorded using GPS [15,16].

### Population size estimation and genetic diversity analysis

654 elephant dung samples were collected from December 2003 to September 2007 (over 100 samples each year) from five regions, including Mengla, Shangyong and Mengyang in Xishuangbanna, Nangunhe in Lincang and Simao in Pu'er. Up to 5grams of dung was collected for each sample peeled from outer layer, and stored in ≥75% ethanol. New gloves and collection tubes were used for collecting each sample to avoid potential cross contamination between different samples. All samples were stored at ambient temperature and transported to the laboratory within eight weeks of collection. The locations of all the samples were recorded via GPS and mapped using Arcview3.2 (http://www.esri.com/software/arcgis/arcview).

DNA extraction was conducted twice for each fecal sample following previously described protocols [18]. Prior to this study, only five microsatellite markers for Asian elephants (EMX-1, EMX-2, EMX-3, EMX-4, and EMX-5) and 40 markers for African elephants (*Loxodonta africana* and *L*. *cydotis*) had been published[19–23]. We tested all of the published markers in our fecal samples and were able to optimize nine markers (EMX-1, EMX-2, EMX-5, LafMS09, LafMS10, FH60, FH94, LA2, and LA3), achieving consistent and successful amplification [24]. Touch-down PCR procedures were conducted for each locus modified from Fernando et al. [19], Nyakaana et al. [23], Comstock et al. [21], Eggert et al. [22] and Cai et al. [24] (see details in S1 Table). To reduce the uncertainty potentially introduced by the trace DNA, each DNA sample was amplified twice using the 9-microsatellite set. A total of four repeat amplifications of each fecal sample were used for cross-validation in genotyping. GeneScanTM-500LIZ (Applied Biosystems) was added to the diluted PCR product to determine the allele size. Fragment sizes were obtained using an Applied Biosystems 3730 and scored using GeneMapper version 3.7 (Applied Biosystems). Allele numbers, allele frequencies, private alleles, and observed and expected heterozygosities were calculated for each locus and each population using GenAlEx 6.1 [25]. DNA-based mark-recapture CAPWIRE software was used to estimate population size [26]. Relative to conventional mark-recapture statistics, the Even Capture Probability Model (ECM) and Two Innate Rates Model (TIRM) in the CAPWIRE were used to handle data inferred from multiple observations of the same individuals. We first computed maximum likelihood estimates of population size using individuals’ capture times and then chose the most suitable model by using the Likelihood Ratio Test (LRT). To obtain the 95% confidence intervals (CI) for the population size estimate, 1,000 parametric bootstraps were performed under the chosen model.

A fragment of mitochondrial DNA (mtDNA), including the 3′ end of the cytochrome b gene, the transfer RNAs for threonine and proline, and partial control region, the latter of which included the hyper variable left domain of the d-loop [19,24,27–29], was amplified using primers MDL3 and MDL5 [30] to assess the mitochondrial DNA diversity of elephant populations in China. The statistical parsimony network was created using TCS ver1.21 [31], combining the haplotypes of present study with previously published haplotypes (GenBank accession Nos. AY245802—AY245827, AY245538, AY365432–365433, and AY589512–589516). The pattern of microsatellite-based population structure was examined using the individual assignment test of the program Structure 2.3.1 [32]. This program used Bayesian inference to generate posterior probabilities of assignment for each individual to a number of genetic clusters (K), which were characterized by a set of allele frequencies at each locus. Ten independent runs of 15 million MCMC iterations by assuming admixture and correlated allele frequencies were conducted for K = 1 to 5 each, with the first 1.5 million iterations discarded as a precedent burn-in. The optimal K value was calculated following Evanno *et al*. [33].

### Habitat spatial analysis and corridor planning

3S technology (Geographic Information System [GIS], Global Positioning System [GPS], and Remote Sensing [RS]) was used to analyze the data obtained from both field surveys and satellite images. Landsat images of elephant range area from the years 1975, 1990, 2005, and 2009 in the Worldwide Reference System (WRS) were used to analyze vegetation cover changes based on the transition matrix method. We used the 90-m resolution Digital Elevation Model (DEM) derived from Shuttle Radar Topography Mission (SRTM) data to generate a 10-m DEM of our study area, given our plot size, through ordinary Kriging Interpolation processes [34]. Elevation, slope, and aspect raster were directly extracted from the interpolated DEM using the Spatial Analyst tools in ArcGIS 9 (ESRI Ltd., CA, USA). Ecological Niche Factor Analysis (ENFA) was applied with the Software Biomapper 4.0 (URL: http://www2.unil.ch/biomapper) to analyze the suitability of elephant habitats across the entire study area [35]. Based on Habitat Suitability Index (HSI) and Boyes Index Curve of cross-validation in the ENFA model, we divided the habitat of the Asian elephant in into different categories: unsuitable, marginal, suitable and optimal habitat [36].

Vanderploge and Scavia’s selectivity index was used to evaluate the habitat selection of elephants according to different environmental parameters [5,15,16]. Based on the interpreted remote sensing images, 32 transects in Nangunhe, 30 transects in Mengyang, 20 transects in Mengla, 41 transects in Shangyong, and 21 transects in Pu’Er were defined, ensuring that all vegetation types could be traversed within the elephant range areas [5,6,10,15,16]. Plots (20m×20m) were sampled at 2–3 km intervals along each transect. Within each plot, latitude, longitude, vegetation type, gradient, orientation, and location were recorded, as well as evidence of elephant activities, such as footprints, dung piles, grubbing, and foraging [12]. Transect surveys were conducted from December 1999 to January 2009, and 4,015 plots were sampled in total.

The research based on fecal DNA lab studies and behavioral observation in the field. The research did not have any animal welfare problem to those elephants during the research period. The corresponding author LZ is a member of the ethic committee of the International Union of Biological Sciences. Although there is not ethics committee in the Beijing Normal University during the study period, the corresponding author is leading a research professor group to develop an animal research protocol for the future study now.

Yunnan Forestry Department approved our research in all elephant ranges in China including Lincang, Pu'er and Xishuangbanna (YFD-BNU 1999, 2003, 2007). MOUs signed between Beijing Normal University and Xishuangbanna National Nature Reserve management authority, and with Nangunhe National Nature Reserve management authority. All field studies did not have any interaction with endangered or protected animals. An ethic committee of Xishuangbanna National Nature Reserve was consulted regarding interviewing the villagers. We only interviewed those villagers who agreed to provide elephant information to the nature reserve's community monitoring network. No any identifying or demographic information was obtained from the villagers. The villagers would be asked whether they were willing to answer our questions, and they could decide to refuse to answer any question during the interview. The Xishuangbanna National Nature Reserve signed and renewed agreements with those villagers working for the community monitoring network annually.

## Results and Discussion

### Population estimates and genetic diversity

Using traditional transects surveys in the field, combined with morphological identification of individuals from photos obtained over the past decade, we estimated the elephant population number in China to be between 221 and 245 (Table 1).

**Table 1:** Estimated sizes of Asian elephant populations in China over time.

Region/Year	1976	1983	1997	2003	2006	2009	2014
Xishuangbanna							
Mengla	37	23		14–17	30	25–32	35–40
Mengyang	26	130	115–137	80–100	46–69	46–63	17–20
Shangyong	38	60	50–60	90–100	60–80	68	53–58
Pu’Er							
Simao	7		5	5	15	15–23	39–47^§^
Lancang				12	9–12	13–17	15^¶^
Jiangcheng							42^‡^
Lincang							
Nangunhe	22	12	18	18	18–23	18–23	20–23
Dehong							
Yingjiang	16			0	0	0	0
Total	146^1^	225^2^	201–233^3^	207–253^4^	165–213^5^	177–216^6^	221–245

^§^ Seasonal migrating population between Simao and Mengyang

^¶^ Seasonal migrating population between Simao and Menghai

^‡^ Seasonal migrating population between Jiangcheng and Mengyang

Data sources:

^1^ Ref. 2

^2^ Ref. 30

^3^ Ref. 13

^4^ Ref. 4

^5^ Ref. 11

^6^ Ref. 3

A total of 479 DNA samples (73.25% of the total dung samples) were successfully extracted and amplified. The mean number of alleles per population ranged from 2.33 (SM) to 3.67 (ML and MY). Expected heterozygosity varied from 0.3167 (SM) to 0.4198 (ML), and observed heterozygosity varied from 0.3565 (NGH) to 0.4132 (ML) (S2 Table). In general, genetic diversity was lower than in other Asian elephant populations [37–38]. All samples in NGH preserved population-based private alleles. ML (28.1%) and SY (27.1%) samples preserved similar levels of private alleles; however, few private alleles were observed in MY (5.4%) and SM (0%) due to their high shared-allele frequency. The locus LafMS09 deviated from Hardy-Weinberg equilibrium with consistently null alleles in all population samples; it was therefore excluded from Structure analyses. All eight remaining loci were in linkage equilibrium. In total, 178 unique genotypes were identified based on the combined microsatellite set, 69 of which were recaptured at least twice. The recapture probability differed significantly among individuals as indicated by the likelihood ratio test. Thus, the TIRM was selected as the best-fit estimation model, yielding a total population size estimate of Asian elephants in China of 186 (95% CI: 178–192). The smallest population is NGH (point estimate: 28, 95% CI: 24–32), and the largest population is Xishuangbanna (point estimate: 94, 95% CI: 87–116).

**Fig 2:**
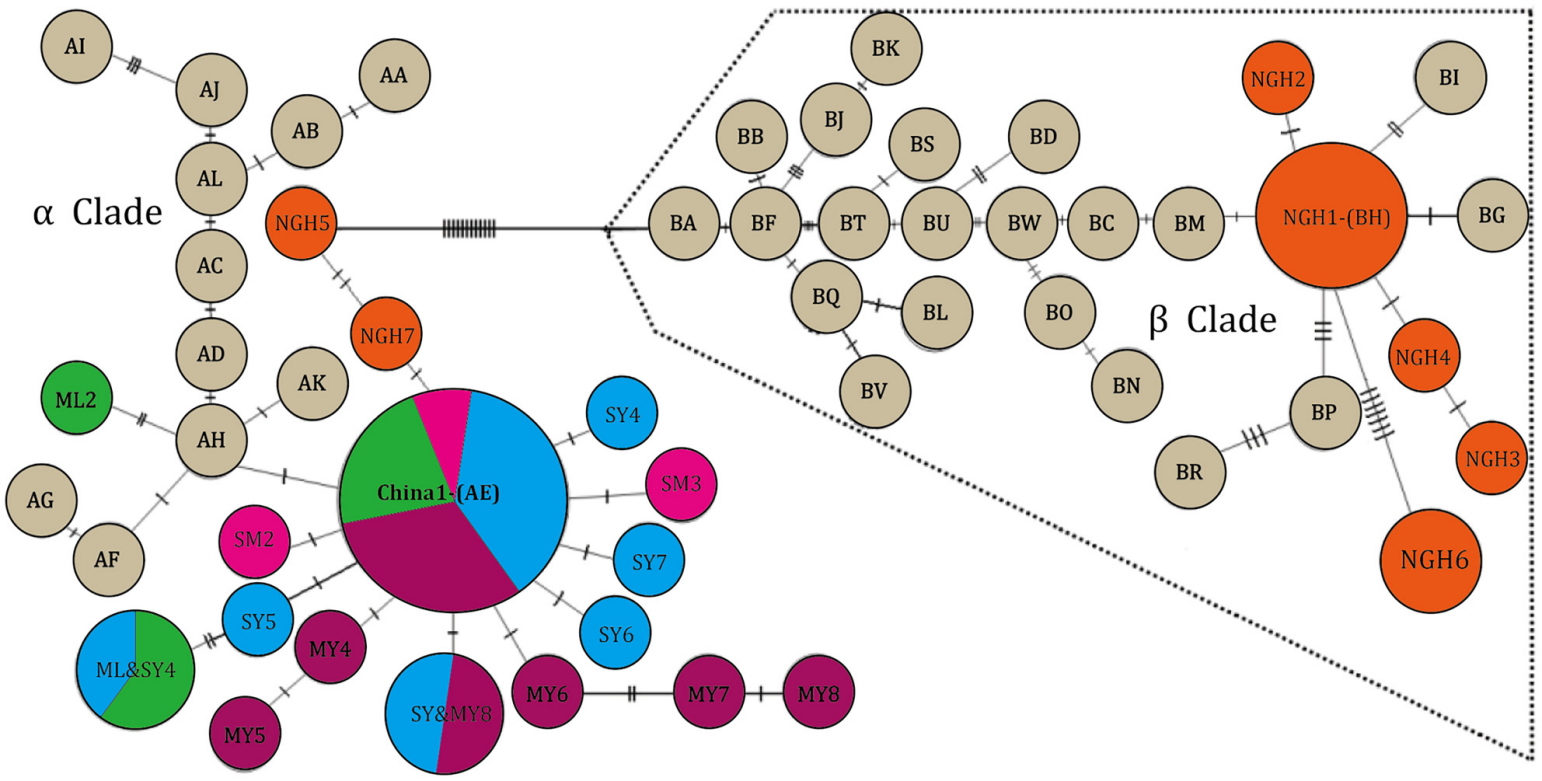
Statistical parsimony network of mtDNA haplotypes based on sequences constructed using the TCS program. AA-AK & BA-BW haplotypes (grey) were from refs.34–35; all others were from present study, Abbreviations of haplotype names—NGH: Nangunhe (orange), ML: Mengla (green), SY: Shangyong (blue), SM: Simao/Pu’er (pink), MY: Mengyang (purple).

A 556-bp fragment of mtDNA was sequenced from 178 genotype individuals. MtDNA diversity in the ML, MY, SM and SY populations was lower than in the other South and Southeast Asian populations, but the NGH population had similar diversity as others (Table 2). Twenty-four haplotypes were identified at 36 variable sites, with 28 parsimony-informative sites and 8 singleton variable sites. One haplotype (designated China 1) carried by 114 individuals was found across four conservation areas (Mengla, Shangyong, Mengyang, and Simao/Pu’Er), corresponding to haplotype AE of Vidya *et al*. [28]. There was no haplotype shared between Nangunhe and the other populations in China, but one (NGH1) shared with the haplotype (BH) in South Asia [28]. An additional 22 haplotypes were reported in Asian elephants the first time compared to previous studies [27–28]. Two highly divergent mtDNA clusters were identified in the Chinese populations (Fig 2) that were consistent with the α and β clusters of Asian elephants descripted by Fenando *et al*. [29]. The Nangunhe population maintained both haplotypes from the α and β cluster and connected the two haplogroups. And the other four populations were grouped into the α cluster.

**Table 2:** Genetic diversity indices of Asian elephants based on mtDNA sequences.

Conservation areas & Geographical unit	Estimated elephant population size	Sample size	Nucleotide diversity (Pi)	Haplotype diversity (Hd)
Shangyong (SY)	60–68	59	0.00140±0.00023	0.528±0.075
Mengla (ML)	25–32	32	0.00126±0.00052	0.337±0.103
Mengyang (MY)	50–82	55	0.00134±0.00061	0.414±0.083
Xishuangbanna	-	154	0.00136±0.00038	0.447±0.050
Simao/Pu’er(SM)	28–30	8	0.00091±0.00036	0.464±0.180
Nangunhe (NGH)	18–23	24	0.00826±0.00206	0.551±0.114
China	178–192	178	0.00726±0.0019	0.579±0.043
Bhutan	250–500	13	0.00652	0.601
India	22,800–32,400	6	-	-
Bhutan-India	-	19	0.00476	0.486
Laos	780–1,200	14	0.00471	0.698
Vietnam	70–100	4	0.00195	0.833
Laos-Vietnam	-	18	0.00431	0.745
Mainland SE Asia	-	37	0.00489	0.758
Northern Sri Lanka	-	18	0.00403	0.758
Mid-lat. Sri Lanka	-	25	0.00453	0.764
Southern Sri Lanka	-	38	0.01289	0.687
Sri Lanka	2,100–3,000	81	0.01643	0.855
Southern India	10,300–17,400	226	0.0036±0.0022	0.436±0.0289
Asia	36,790–51,160	118	0.0176	0.873

Data from Bhutan-India, Laos-Vietnam, Mainland, Sri Lanka, and Asia were cited from Ref. 19; data from Southern India were cited from Refs. 27–29.

**Fig 3:**
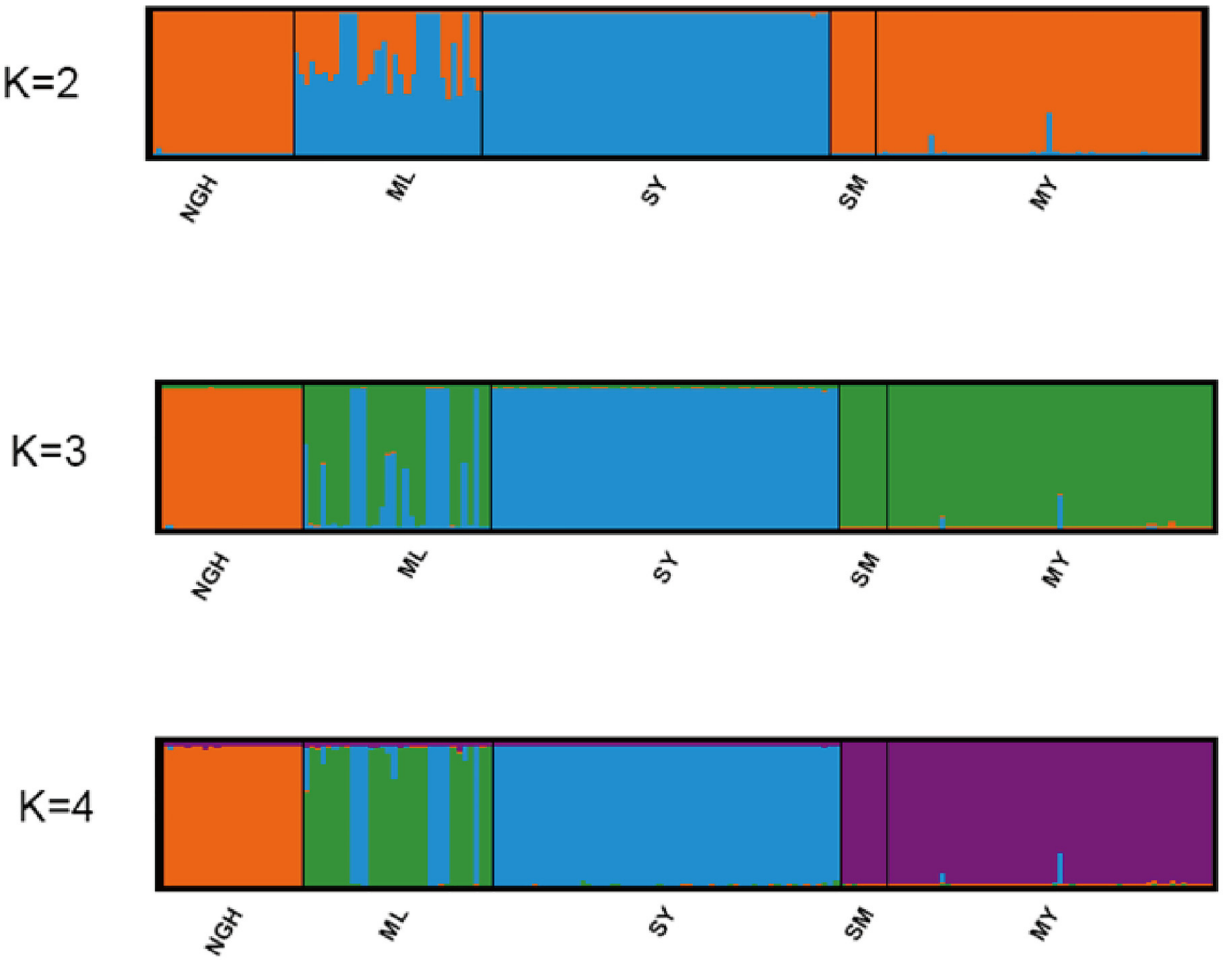
Posterior assignment probabilities of Asian elephants in China determined by a Bayesian algorithm implemented in the program STRUCTURE v2.3.1 [32]. K = 4 was selected as the most likely number of clusters under the ln K and K difference comparison according to Evannoet al. [33]. Abbreviation of population names—NGH: Nangunhe, ML: Mengla, SY: Shangyong, SM: Simao/Pu’er, MY: Mengyang.

In our ten independent replicates of the Bayesian Structure analysis for the estimation of K, values of LnP(D) sharply increased as K increased from 1 to 4, and delta K had a prominent peak at K = 4. Under the optimal cluster number (K = 4), the elephant populations separated into four clusters (Fig 3). Individuals from Nangunhe were significant divergent from other populations. Members of the Shangyong population formed a cluster together with some individuals from Mengla, whereas other Mengla individuals preserved a distinct genetic background. The Mengyang population formed another cluster along with individuals from Simao/Pu’Er. Considering the genetic clusters of mtDNA and microsatellites together, the results suggested that the Asian elephants in China should be managed as either two (Nangunhe and other four populations) or four (Nangunhe, Mengyang and Simao, Mengla, and Shangyong) separate conservation units, and different protection strategies should be developed to promote their long-term survival.

Since 1960, much of the original forests in Mengla County had been cut down to plant rubber trees, and since 1980, the affected area had continued to grow. Due to the rapid increase in the price of rubber in the international market in recent years, more original forest had been destroyed to plant rubber trees in Xishuangbanna. This had ecologically isolated Mengxing-Xiangming-Yiwu, including the large and small Kongming Hills and Yao areas, and formed geographical barriers between Shangyong-Mengla (SY-ML) and Pu’Er-Mengyang (SM-MY), driving elephants to withdraw their traditional home ranges. There had been no migration between elephant populations from the two areas over the past several decades. Genetic drift among wild Asian elephant populations is expected to proceed slowly because elephants are large mammals with large territories, long reproductive cycles (the observed reproductive age of females was typically 18–20 years old, with the youngest age being 14–15 years), and low birth rates (1 offspring every 2.5–8 years per female, on average). Over the past five decades, extensive removal of forests and reclamation of farmland had fragmented elephant habitat. The original forests had contracted and became fragmented by rubber plantations and farmland, resulting in a lack of gene flow between the two isolated populations. However, as these events were recent, no significant genetic differences and no obvious genetic divergence had yet arisen between the two populations.

The optimal solution for enhancing gene flow among the geographically isolated populations is to establish ecological corridors. The feasibility and necessity of building ecological corridors among the Mengyang (MY), Shangyong (SY) and Mengla (ML) nature reserves had been evaluated [15,16]. Such corridors are expected to secure elephant migrating routes and facilitate gene flow among those isolated populations.

We also detected no shared mtDNA haplotypes between the Xishuangbanna and Nangunhe populations. In the 1980s, the Nangunhe population was likely contiguous with those in Myanmar; however, because of the subsequent severe habitat destruction, no gene flow has been possible since 1997. Therefore, the Nangunhe elephants are now an isolated population composed of 18–21 individuals. The F_ST_ test of haplotype frequencies revealed a significant difference between the Nangunhe population and the other four geographic populations in Pu’Er and Xishuangbanna (Table 2). The Nangunhe population and the Xishuangbanna population carried mtDNA belonging to two distinct clades (β and α, respectively).

### Use of ENFA analysis to evaluate Asian elephant habitats

In 1996, five elephants migrated from Mengyang of Xishuangbanna and settled in Simao. Since then, more elephants had migrated from Xishuangbanna to Pu’Er due to the infrastructure constructions, such as the Si-Xiao highway and the Nuozhadu dam, and the expansion of tea and rubber plantations. Currently, there are 71–75 elephants living in Pu’Er, representing eight populations. Using Ecological Niche Factor Analysis (ENFA), we found that the marginality value of the Asian elephant in Pu’Er was 0.992, which was very high and approached 1. This result indicated a deviation of their preferable niche from the average value of the background environment and suggested that the selection of environmental variables was not random. The tolerance value was 0.315, indicating that the niche breadth of the Asian elephant was narrow and that elephant survival was restricted by environmental conditions.

Based on Habitat Suitability Index (HSI) from the ENFA model, we divided the habitat of the Asian elephant in into four different categories: unsuitable habitat (0<HSI≤12), marginal habitat (12<HSI≤38), optimal habitat (38<HSI≤64) and suitable habitat (65<HSI≤100).

The results showed that Pu’Er provided 409.32 km^2^ of optimal elephant habitat, 574.32 km^2^ suitable habitat, 2,909.48 km^2^ marginal habitat and 38,722.32 km^2^ unsuitable habitat. The unsuitable habitat accounted for 90.86% of the total area, while optimal habitat regions only 0.96% of the total area. According to the habitat distribution map, both optimal and suitable habitats, which comprised small areas, occurred in all counties of Pu’Er, except Ximeng County. The largest area of optimal habitat (128.48km^2^, 31.39% of the total optimal habitat) and suitable habitat (149.32 km^2^, 26% of the total suitable habitat) were found in Jiangcheng County. Landscape analysis showed that optimal, suitable, and marginal habitats all suffered from severe fragmentation, low connectivity, and high human disturbance.

Based on these results, we recommend that new protected areas be established as soon as possible in the range of the Asian elephant, coupled with effective and legal protection measures in Pu’Er. Additionally, it is important to establish ecological corridors to link all the small family groups in the region and to enhance the connection with those elephants in Xishuangbanna.

Based on the separated vegetation and administrative boundaries, we divided the Xishuangbanna Prefecture into two parts: Jinghong-Menghai and Shangyong-Mengla. The marginality values of the two parts were both greater than 1, and the tolerance values were both approximately 0.5, suggesting that the elephants were selective with respect to environmental variables, although they had some tolerance and adaptability to the environment. Of all of the environmental factors, vegetation, particularly the distribution of bamboo, was the primary factor affecting habitat quality in both areas, indicating that food was the most important factor influencing habitat selection by the Asian elephant.

Currently, Xishuangbanna provided 952.75km^2^ of optimal elephant habitat, 1,965.25km^2^ of suitable habitat, and 2,589.25km^2^ of marginal habitat; these values were predicted from all of the distribution points of all elephants, including both farmland and non-farmland. In contrast, unsuitable habitats occupied 71.2% of Xishuangbanna. We found that 30.7% of suitable habitats were farmland, which was the result from the software “Biomapper” analysis based on those plots with elephant occupancy inside farmlands. Analysis of the distribution points within non-farmland areas alone identified 436.75km^2^ of optimal habitat, 1,443.75km^2^ of suitable habitat, and 974.75km^2^ of marginal habitat. These results showed that the amount of suitable habitat decreased sharply after farmland sites were excluded. Farmland had always been regarded as components of the elephants’ foraging habitat and migration path, rather than true suitable habitat. However, loss and fragmentation of the elephant habitat by degradation and establishment of rubber plantations had resulted in crop raiding and the dispersion of elephants out of that nature reserves, leading to severe human-elephant conflicts.

The Asian elephant population was distributed over three main areas in Xishuangbanna Prefecture: Mengyang, Mengla, and Shangyong. Suitable habitats outside of the nature reserves contained the elephants’ migration routes. This result suggested that ecological buffers should be established in these key regions to connect the Mengyang-Lancang (Corridor 1), Mengyang-Simao (Corridor 2), Mengyang-Jiangcheng (Corridor 3) and Mengla-Shangyong (Corridor 4) habitats as elephants migrate through the corridors (see Fig 1 for details). In addition, as suggested by our genetic analyses, a corridor restoration program to reconnect the isolated populations was critical to the survival of the Asian elephant in China. Therefore, we recommended that the reserve management department establish these corridors as early as possible to prevent further habitat fragmentation, mitigate the pressure of human activities, and improve gene flow among the populations.

## Supporting Information

S1 Table PCR protocols for the nine microsatellites loci. (DOCX)

S2 Table Genetic diversity of 9 microsatellites of Asian Elephants in China (Na: Observed Number of Alleles; NE: Effective Number of Alleles; I: Shannon Information Index; Nei's: Nei's Gene Diversity; HO: Observed Heterozygosity; HE: Expected Heterozygosity; p: Percentage of Polymorphic loci; PIC: Polymorphic Information Content). (DOCX)
