# Supplementary material for: Asian Elephants in China: Estimating Population Size and Evaluating Habitat Suitability
Source: PLoS One. 2015 May 19;10(5):e0124834. doi: 10.1371/journal.pone.0124834 (PMC4438002; doi:10.1371/journal.pone.0124834)
Supplement: S1 Table — (DOCX) [file pone.0124834.s001.docx]

S1 Table. PCR protocols for the nine microsatellites loci

| **Locus** | **Step** | **T (℃)** | **Time** |
| --- | --- | --- | --- |
| EMX1, EMX2, EMX5, LafMS09 | Denaturation | 95 | 5 min |
|  | 10 cycles; 1℃ drop for each cycle until 59℃ | 95 | 30 sec |
|  |  | 68 | 30 sec |
|  |  | 72 | 30 sec |
|  | 30 cycles | 95 | 30 sec |
|  |  | 58 | 30 sec |
|  |  | 72 | 30 sec |
|  | Extension | 72 | 15 min |
| FH60, FH94, LA2, LA3 | Denaturation | 95 | 5 min |
|  | 10 cycles; 1℃ drop for each cycle until 53℃ | 95 | 30 sec |
|  |  | 62 | 30 sec |
|  |  | 72 | 30 sec |
|  | 30 cycles | 95 | 30 sec |
|  |  | 52 | 30 sec |
|  |  | 72 | 30 sec |
|  | Extension | 72 | 15 min |
| LafMS10 | Denaturation | 95 | 5 min |
|  | 5 cycles; 1℃ drop for each cycle until 56℃ | 94 | 30 sec |
|  |  | 60 | 1 min 30 sec |
|  |  | 72 | 1 min |
|  | 30 cycles | 94 | 30 sec |
|  |  | 55 | 1 min 30 sec |
|  |  | 72 | 1 min |
|  | Extension | 60 | 30 min |
