# Supplementary material for: Asian Elephants in China: Estimating Population Size and Evaluating Habitat Suitability
Source: PLoS One. 2015 May 19;10(5):e0124834. doi: 10.1371/journal.pone.0124834 (PMC4438002; doi:10.1371/journal.pone.0124834)
Supplement: S2 Table — (DOCX) [file pone.0124834.s002.docx]

S2 Table. Genetic diversity of 9 microsatellites of Asian Elephants in China (*Na*: Observed Number of Alleles; *N_E_*: Effective Number of Alleles; *I*: Shannon Information Index; Nei's: Nei's Gene Diversity; *H_O_*: Observed Heterozygosity; *H_E_*: Expected Heterozygosity; *p*: Percentage of Polymorphic loci; *PIC*: Polymorphic Information Content).

| **Locus** | **Parameter** | **Geographical Populations*** | | | | |
| --- | --- | --- | --- | --- | --- | --- |
|  |  | **NGH** | **ML** | **SY** | **SM** | **MY** |
| EMX-1 | *Na* | 2.0000 | 3.0000 | 4.0000 | 2.0000 | 3.0000 |
|  | *N_E_* | 1.1327 | 1.2511 | 1.2324 | 1.2800 | 1.1786 |
|  | *I* | 0.2338 | 0.4199 | 0.4222 | 0.3768 | 0.3117 |
|  | *H_O_* | 0.1250 | 0.2188 | 0.2034 | 0.2500 | 0.1636 |
|  | *H_E_* | 0.1197 | 0.2039 | 0.1902 | 0.2333 | 0.1530 |
|  | *Nei's* | 0.1172 | 0.2007 | 0.1886 | 0.2188 | 0.1516 |
|  | *PIC* | 0.1103 | 0.1910 | 0.1814 | 0.1948 | 0.1425 |
| EMX-2 | *Na* | 2.0000 | 2.0000 | 1.0000 | 2.0000 | 2.0000 |
|  | *N_E_* | 1.1327 | 1.0981 | 1.0000 | 1.1327 | 1.0370 |
|  | *I* | 0.2338 | 0.1892 | 0.0000 | 0.2338 | 0.0909 |
|  | *H_O_* | 0.1250 | 0.0938 | 0.0000 | 0.1250 | 0.0364 |
|  | *H_E_* | 0.1197 | 0.0908 | 0.0000 | 0.1250 | 0.0360 |
|  | *Nei's* | 0.1172 | 0.0894 | 0.0000 | 0.1172 | 0.0357 |
|  | *PIC* | 0.1103 | 0.0854 | 0.0000 | 0.1103 | 0.0351 |
| EMX-5 | *Na* | 5.0000 | 5.0000 | 1.0000 | 2.0000 | 6.0000 |
|  | *N_E_* | 1.9965 | 1.3875 | 1.0000 | 1.1327 | 2.1132 |
|  | *I* | 0.9534 | 0.6159 | 0.0000 | 0.2338 | 1.0413 |
|  | *H_O_* | 0.2917 | 0.1875 | 0.0000 | 0.1250 | 0.4364 |
|  | *H_E_* | 0.5098 | 0.2837 | 0.0000 | 0.1250 | 0.5316 |
|  | *Nei's* | 0.4491 | 0.2793 | 0.0000 | 0.1172 | 0.5268 |
|  | *PIC* | 0.4486 | 0.2667 | 0.0000 | 0.1103 | 0.4791 |
| LafMS09 | *Na* | 2.0000 | 2.0000 | 2.0000 | 2.0000 | 2.0000 |
|  | *N_E_* | 1.9459 | 1.9922 | 1.9908 | 2.0000 | 1.9836 |
|  | *I* | 0.6792 | 0.6912 | 0.6908 | 0.6931 | 0.6890 |
|  | *H_O_* | 0.8333 | 0.9375 | 0.9322 | 1.0000 | 0.8727 |
|  | *H_E_* | 0.4965 | 0.5060 | 0.5020 | 0.5333 | 0.5004 |
|  | *Nei's* | 0.4861 | 0.4980 | 0.4977 | 0.5000 | 0.4959 |
|  | *PIC* | 0.3680 | 0.3740 | 0.3738 | 0.3750 | 0.3729 |
| FH60 | *Na* | 3.0000 | 6.0000 | 5.0000 | 2.0000 | 3.0000 |
|  | *N_E_* | 2.4253 | 3.5930 | 2.4015 | 1.9692 | 2.1708 |
|  | *I* | 0.9673 | 1.4088 | 1.0247 | 0.6853 | 0.8663 |
|  | *H_O_* | 0.9583 | 0.9062 | 0.7797 | 0.8750 | 0.8364 |
|  | *H_E_* | 0.6002 | 0.7331 | 0.5886 | 0.5250 | 0.5443 |
|  | *Nei's* | 0.5877 | 0.7217 | 0.5836 | 0.4922 | 0.5393 |
|  | *PIC* | 0.5072 | 0.6732 | 0.5041 | 0.3711 | 0.4474 |
| FH94 | *Na* | 5.0000 | 5.0000 | 7.0000 | 3.0000 | 6.0000 |
|  | *N_E_* | 1.7888 | 1.5445 | 1.9169 | 1.2929 | 1.6947 |
|  | *I* | 0.8871 | 0.7618 | 0.9135 | 0.4634 | 0.8615 |
|  | *H_O_* | 0.4167 | 0.3125 | 0.6441 | 0.2500 | 0.4364 |
|  | *H_E_* | 0.4504 | 0.3581 | 0.4824 | 0.2417 | 0.4137 |
|  | *Nei's* | 0.4410 | 0.3525 | 0.4783 | 0.2266 | 0.4099 |
|  | *PIC* | 0.4115 | 0.3366 | 0.4211 | 0.2146 | 0.3861 |
| LA3 | *Na* | 3.0000 | 3.0000 | 2.0000 | 3.0000 | 3.0000 |
|  | *N_E_* | 1.4527 | 1.8568 | 1.5454 | 2.0317 | 1.4060 |
|  | *I* | 0.5480 | 0.8136 | 0.5378 | 0.8305 | 0.4958 |
|  | *H_O_* | 0.2083 | 0.4062 | 0.2203 | 0.5000 | 0.2000 |
|  | *H_E_* | 0.3183 | 0.4688 | 0.3559 | 0.5417 | 0.2914 |
|  | *Nei's* | 0.3116 | 0.4614 | 0.3529 | 0.5078 | 0.2888 |
|  | *PIC* | 0.2744 | 0.4168 | 0.2906 | 0.4277 | 0.2520 |
| LafMS10 | *Na* | 3.0000 | 4.0000 | 2.0000 | 1.0000 | 3.0000 |
|  | *N_E_* | 1.1350 | 2.0378 | 1.1447 | 1.0000 | 1.1592 |
|  | *I* | 0.2736 | 0.8657 | 0.2479 | 0.0000 | 0.3088 |
|  | *H_O_* | 0.0417 | 0.0938 | 0.0678 | 0.0000 | 0.0364 |
|  | *H_E_* | 0.1215 | 0.5174 | 0.1275 | 0.0000 | 0.1386 |
|  | *Nei's* | 0.1189 | 0.5093 | 0.1264 | 0.0000 | 0.1374 |
|  | *PIC* | 0.1151 | 0.4306 | 0.1184 | 0.0000 | 0.1325 |
| LA2 | *Na* | 4.0000 | 3.0000 | 5.0000 | 4.0000 | 5.0000 |
|  | *N_E_* | 1.2958 | 2.5441 | 3.0751 | 1.9692 | 1.3838 |
|  | *I* | 0.5032 | 1.0077 | 1.3050 | 0.9507 | 0.6168 |
|  | *H_O_* | 0.2083 | 0.5625 | 0.7627 | 0.3750 | 0.2182 |
|  | *H_E_* | 0.2332 | 0.6166 | 0.6806 | 0.5250 | 0.2799 |
|  | *Nei's* | 0.2283 | 0.6069 | 0.6748 | 0.4922 | 0.2774 |
|  | *PIC* | 0.2190 | 0.5335 | 0.6273 | 0.4582 | 0.2814 |
| Mean | *Na* | 3.2222 | 3.6667 | 3.2222 | 2.3333 | 3.6667 |
|  | *N_E_* | 1.5895 | 1.9228 | 1.7008 | 1.5343 | 1.5697 |
|  | *I* | 0.5866 | 0.7526 | 0.5713 | 0.4964 | 0.5869 |
|  | *H_O_* | 0.3565 | 0.4132 | 0.4011 | 0.3889 | 0.3596 |
|  | *H_E_* | 0.3299 | 0.4198 | 0.3252 | 0.3167 | 0.3210 |
|  | *Nei's* | 0.323 | 0.4132 | 0.3225 | 0.2969 | 0.3181 |
|  | *PIC* | 0.2849 | 0.3675 | 0.2796 | 0.2513 | 0.2810 |

* NGH: Nangunhe; ML: Mengla; SY: Shangyong; SM: Simal; MY: Mengyang.
